# A survey on socioeconomic determinants of diabetes mellitus management in a lower middle income setting

**DOI:** 10.1186/s12939-016-0363-3

**Published:** 2016-05-04

**Authors:** Ambepitiyawaduge Pubudu De Silva, Sudirikku Hennadige Padmal De Silva, Rashan Haniffa, Isurujith Kongala Liyanage, Kosala Saroj Amarasiri Jayasinghe, Prasad Katulanda, Chandrika Neelakanthi Wijeratne, Sumedha Wijeratne, Lalini Chandika Rajapakse

**Affiliations:** Department of Community Medicine, Faculty of Medicine, University of Colombo, No. 25, P.O. Box 271, Kynsey Road, Colombo 08, Sri Lanka; Centre for Tropical Medicine, University of Oxford, Oxford, UK; Department of Para Clinical Sciences, Faculty of Medicine, General Sir John Kotelawala University, Colombo, Sri Lanka; Department of Clinical Medicine, Faculty of Medicine, University of Colombo, Colombo, Sri Lanka; Department of Obstetrics & Gynaecology, Faculty of Medicine, University of Colombo, Colombo, Sri Lanka

**Keywords:** Diabetes mellitus complications, Diabetes mellitus management, Socioeconomic determinants

## Abstract

**Background:**

Information on socioeconomic determinants in the management of diabetes mellitus is scarce in lower middle income countries. The aim of this study is to describe the socioeconomic determinants of management and complications of diabetes mellitus in a lower middle income setting.

**Methods:**

Cross sectional descriptive study on a stratified random sample of 1300 individuals was conducted by an interviewer administered questionnaire, clinical examinations and blood investigations. A single fasting venous blood sugar of ≥126 mg/dl was considered diagnostic of new diabetics and poor control of diabetes mellitus as HbA_1_C > 6.5 %.

**Results:**

There were 202 (14.7 %) with diabetes mellitus. Poor control was seen in 130 (90.7 %) while 71 (49.6 %) were not on regular treatment. Highest proportions of poor control and not on regular medication were observed in estate sector, poorest social status category and poorest geographical area. The annual HbA_1C_, microalbuminuria, retinal and neuropathy examination were performed in less than 6.0 %. Social gradient not observed in the management lapses. Most (76.6 %) had accessed private sector while those in estate (58.1 %) accessed the state system.

The microvascular complications of retinopathy, neuropathy and microalbuminuria observed in 11.1 %, 79.3 % and 54.5 % respectively. Among the macrovascular diseases, angina, ischaemic heart disease and peripheral arterial disease seen in 15.5 %, 15.7 % and 5.5 % respectively. These complications do not show a social gradient.

**Conclusions:**

Diabetes mellitus patients, irrespective of their socioeconomic status, are poorly managed and have high rates of complications. Most depend on the private healthcare system with overall poor access to care in the estate sector.

## Background

Diabetes mellitus is a global public health problem with a majority in developing countries [1]. In 2008 the age-standardised prevalence of diabetes mellitus for men and women was 9.8 % (8.6–11.2) and 9.2 % (8.0–10.5) respectively, with 40 % of these residing in China and India [1]. The projected increase over the period from 1995-2025 for developed and developing countries was 42 % and 170 % respectively [2]. In 2025 it is expected that 75 % of diabetics will reside in developing countries [3].

Diabetes mellitus is a progressive disease requiring effective lifelong medical care for the prevention of secondary and tertiary complications. The optimal control of blood glucose has clearly demonstrated a significant decrease in the development of complications [4]. Control of diabetes mellitus requires the combination of treatment and preventive action, taking into account biological and health behavioural factors, health service responsiveness, and socioeconomic conditions [5].

The relationship between socioeconomic positions and the prevalence, management and complications of diabetes mellitus are well known in High Income Countries (HICs) with low socioeconomic status associated with unhealthy behaviours, poor access to care, deficient processes of care and high rate of diabetes complications [6–19].

In Low Income Countries (LICs)/Lower Middle Income Countries (LMICs) the socioeconomic factors are mostly explored in relation to the prevalence of diabetes mellitus while little is known on social determinants of complications and management of this condition [3, 20–24]. According to the World Bank LICs are defined as those with a gross national income per capita of $1,045 or less in 2014 while for HICs the figure is $12,736 or more [25].The few studies which investigate the complications and management of diabetes mellitus in low and lower middle income settings are usually restricted to hospital attendees [22, 26, 27]. The available data on complications and management of diabetes mellitus in Sri Lanka are limited to state hospital attendees, excluding many patients who obtain services from the private healthcare sector [28–34]. In Sri Lanka the out-patient care for more than 50 % of the population is provided by the private healthcare sector while in-patient care for more than 90 % is provided by the state healthcare sector [35]. State healthcare in Sri Lanka is free at the point of delivery to all citizens of Sri Lanka. This includes all visits/consultations (out-patient as well as in-patient care including intensive care services and surgical care), medications, investigations and procedures including ambulance transportation/transfers and hospital meals.

Understanding the socioeconomic determinants in the management and complications of diabetes mellitus would help to focus measures to address existing issues related diabetes mellitus [3, 36]. This would further aid to design specific strategies aimed at reducing inequalities and shed more light as to why control measures of diabetes mellitus have failed in South Asian region.

This study is a community based survey to describe the socioeconomic determinants of diabetes mellitus management in a representative sample from a suburban area, in Sri Lanka.

## Methods

A detailed description of the study method is already published [37]. A sample of 1300 individuals between the ages of 35 to 64 years was randomly selected representing the urban, rural and estate sector. The estate sector mainly consists of tea, rubber and coconut plantation sectors and housed to approximately 6 % of Sri Lankans. Compared to urban and rural sectors it is the least resourced and poorest. In this study setting tea and rubber plantation sectors were included.

Data were collected using trained data collectors with validated questionnaires (social status index questionnaire, Rose questionnaire for angina detection and questionnaire to assess neuropathy symptom score & modified neuropathy disability score) [29, 30, 38, 39]. When administering the questionnaire the participants were simply asked whether they received any information regarding the diseases and if so from whom.

All participants were investigated for diabetes mellitus by conducting fasting plasma glucose (FPG) level using a venous blood sample. The blood samples were obtained after an overnight fast of at least 12 hours. The collected blood samples were analysed at the Public Health Laboratory of National Institute of Health Sciences (Kalutara), using Clini Check Plus Mini Analyser. FPG of ≥126mg/dl or those who were currently (within the past four weeks) on insulin/hypoglycaemics were considered as having diabetes mellitus [40].

The Unsatisfactory Basic Needs Index (UBNI) developed by Satharasinghe [41] and the social status index developed by De Silva [39] were utilized as measures of socioeconomic matrices. The UBNI is an area level deprivation index calculated taking into consideration the level of education, occupation, housing conditions (wall, roof and floor), source of lighting and cooking. It has a correlation coefficient of 0.62 with the Headcount ratio [41]. The social status index composed of education, occupation, income, assets and social networking. The reliability revealed a Cohen’s kappa coefficient >0.78 for each item category. The criterion (level of agreement 65 %) and construct validity (Root Mean Square Error of Approximation = 0.056) were satisfactory [39]. The highest level of education attained was recorded. Those who have passed the General Certificate of Education - Ordinary Level examination (which is held at the end of Grade 10) but has failed to complete the General Certificate of Education -Advanced Level examination was grouped as Ordinary Level to Grade 12 (which includes Grade 11 as well). Those who have passed General Certificate of Education -Advanced Level (which was held at the end of Grade 12) was grouped as Advanced Level and above.

All participants (old and new) diagnosed with diabetes mellitus were assessed by the first author who was trained on clinical examinations of diabetic complications and calibrated against a specialist (Cohen’s kappa coefficient 0.7 for diabetic retinopathy). Measures were taken to maintain the quality and accuracy of the data [37].

Diabetic retinopathy was diagnosed by clinical examination. Visual acuity was tested in corrected state, using standard 6 m Snellen chart for each eye. Patients were asked to bring their spectacles for the eye test. Pinhole correction was used when spectacles were not brought. Fundus examination was carried out by direct ophthalmoscopy performed after pupils were dilated. The patients gaze was fixed away from light except when examining the macular areas when they were asked to look directly at the light. Those in the stage of mild nonproliferative retinopathy or above were considered as having diabetic retinopathy. Neuropathy was assessed by a validated neuropathy symptom score and modified neuropathy disability score [30, 33]. A spot urine albumin was measured in the absence of urinary tract infection, with turbidimetric method using Rx Daytona machine. The presence of urine albumin >30mg/l was considered as microalbuminuria. The presence of diabetic retinopathy, neuropathy or microalbuminuria was considered as having microvascular disease.

Ischaemic Heart Disease (IHD) was diagnosed by 12 lead electrocardiography (ECG) using the Minnesota coding system [42]. Evidence of large Q or QS waves, those with complete left bundle branch block, presence of small Q waves, ST segment abnormalities and T wave abnormalities were considered as existence of IHD. Angina was diagnosed using the Rose questionnaire [38] and Peripheral Arterial Disease (PAD) was diagnosed using the ankle-brachial pressure index (ABI) [43]. The ABI of 0.91–1.30 was considered as normal. The presence of IHD, angina or PAD was considered as having macrovascular disease.

Diabetic patients who have discontinued medication for more than seven days, on one or more occasions, during the preceding year, were categorized as those who were not on continuous treatment. Visit to a medical professional (state or private sector) on every month during the preceding year was identified as successful monthly follow-ups [44]. Failure to visit on two or more consecutive months was regarded as failure in monthly follow-ups. Self reported impotence, early ejaculation or late ejaculation was considered as having sexual problems. We also documented self reported hypoglycaemic attacks. The control of diabetes was assessed by the levels of glycosylated haemoglobin [45]. Those with glycosylated haemoglobin of more than 6.5 % were considered as having poor control of diabetes mellitus. The use of Benedict’s solution at home for detection of reducing substance in urine was inquired from the study participants [46].

Data were analysed using STATA 13. Findings were weighted to make a correction for the over sampling of urban and estate sectors. Results were also adjusted for age and sex of the Sri Lankan population. Standard descriptive statistics was performed. All percentages given in the results sections are expressed as weighted values. Chi square was used to compare discrete variables.

Ethics approval was obtained from the Ethics Review Committee of Faculty of Medicine, University of Colombo (EC/08/119). Informed written consent was obtained from the study participants.

## Results

**Fig. 1:**
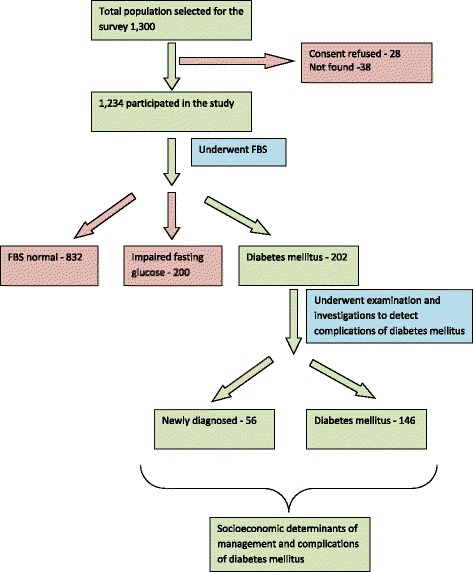
The flow chart of the study

From the 1,300 selected individuals 1,234 (94.9 %) participated (Fig. 1). Our previous publication showed that among the participants who were screened (628 males), 202 (14.7 %) had diabetes mellitus [37]; 22.8 % of diabetics (56 individuals) were newly diagnosed.

Of the 146 (77.2 %) known to have diabetes mellitus, there were 12 (12.1 %) insulin users, and 133 (86 %) on oral medication for diabetes mellitus while one (1.9) was exclusively on diet control. While most (130, 67.1 %) received information on diet from their treating medical doctor, few (21, 13.2 %) received information on the importance of tight control of blood sugar. Table 1 describes management of already diagnosed individuals with diabetes mellitus (n = 146) and the complications among all diabetics (n = 202).

**Table 1:** Management and complications of individuals with diabetes mellitus

Management and complications	Number	Percentage^a^
Place of regular visit for treatment (n = 146)		
State	41	23.4 %
Private	100	76.6 %
Missing	5	
Management activities (n = 146)^b^		
Monthly follow-ups done	53	38.6 %
Following a diabetic diet	16	14.4 %
Diet control	16	14.4 %
Referred to a dietician	4	3.0 %
Diet plan by dietician	1	0.1 %
Referred to an ophthalmology clinic	6	6.6 %
Annual retinal examination	3	4.2 %
Neuropathy examination	0	0.0 %
Frequency of investigations conducted (n = 146)^b^		
Monthly fasting plasma glucose	62	47.2 %
Annual plasma lipids	25	13.9 %
Annual electrocardiogram	7	4.7 %
Annual HbA_1_C	4	2.5 %
Annual urine microalbumin	5	5.2 %
Benedict’s test at home (at least monthly)	7	4.8 %
Capillary blood sugar at home (at least monthly)	5	3.6 %
Complications (n = 202)^b^		
Diabetic retinopathy	30	9.8 %
Neuropathy	159	82.9 %
Microalbuminurea	61	33.8 %
Angina	35	14.4 %
Ischaemic heart disease	29	31.7 %
Peripheral arterial disease	8	5.1 %
Foot ulcer	22	9.0 %
Amputation	2	0.1 %
Sexual problems (among males only, n = 98) (impotence, early/late ejaculation)	43	61.6 %

^a^All percentages were weighted values, ^b^Based on multiple responses

Significant differences (p < 0.05) in the frequency of diabetic retinopathy and ischaemic heart disease were observed between the newly diagnosed (diabetic retinopathy 4.1 %, ischaemic heart disease 11.1 %) and already known diabetes mellitus (diabetic retinopathy 11.5 %, ischaemic heart disease 37.6 %) groups. Almost all complications were higher except neuropathy, among the already known diabetes when compared to the newly diagnosed.

As many as 71 (49.6 %) adults with diabetes mellitus were not on regular medication. Glycaemic control, as defined by HbA_1_C, was poor in 130 (90.7 %) adults. Table 2 describes the distribution of diabetes management aspects by socioeconomic characteristics.

**Table 2:** The distribution of diabetes mellitus (DM) management aspects by socioeconomic characteristics

Socioeconomic characteristic	Newly diagnosed with ^a^DM (n = 56)		Already diagnosed of ^a^DM (146)		Poor Control of ^a^DM (n = 130)		Not on continuous treatment (n = 71)		Monthly follow-ups not done (n = 91)	
	Number	%^b^	Number	%^b^	Number	%^b^	Number	%^b^	Number	%^b^
Sex										
Male	34	27.1 %	64	72.9 %	58	95.8 %	36	55.7 %	48	75.7 %
Female	22	19 %	82	81 %	72	86.5 %	35	44.6 %	43	49.5 %
Age (years)										
35 to 39	5	49.9 %	6	50.1 %	6	100 %	4	51.0 %	5	52.1 %
40 to 44	10	35.2 %	19	64.8 %	17	89.0 %	11	66.0 %	13	56.4 %
45 to 49	13	28.7 %	26	71.3 %	21	80.2 %	13	31.4 %	15	50.4 %
50 to 54	4	0.4 %	36	99.6 %	35	93.5 %	20	65.9 %	25	79.2 %
55 to 59	13	22 %	30	78 %	26	94.0 %	11	29.5 %	15	49.8 %
60 to 64	11	22.7 %	29	77.2 %	25	92.3 %	12	52.9 %	18	65.4 %
Ethnicity										
Sinhalese	40	23.9 %	116	76.1 %	103	91.9 %	59	51.2 %	72	63.6 %
Tamil	11	7.3 %	12	92.7 %	11	99.0 %	6	4.2 %	8	5.6 %
Muslim	5	3.3 %	18	96.7 %	15	67.1 %	6	33.1 %	11	37.0 %
Education										
No schooling	4	2.2 %	2	97.8 %	1	41.0 %	-	-	-	-
Grade 5 or below	9	35.2 %	23	64.8 %	21	99.3 %	9	24.0 %	9	47.6 %
Grade 6 to Grade 10	24	21.3 %	55	78.7 %	52	97.5 %	25	47.4 %	33	52.6 %
Ordinary Level to Grade 12	14	8.3 %	30	91.7 %	26	90.5 %	17	53.0 %	23	73.5 %
Advanced Level and above	4	23.4 %	27	76.6 %	24	93.4 %	17	64.8 %	19	70.8 %
Missing	1		9		3		3		7	
Occupation										
Professional	1	34 %	2	66 %	2	100 %	2	100 %	2	100 %
Technical & clerical	3	3 %	9	97 %	9	100 %	7	79.5 %	8	98.9 %
Vendors and sellers	10	18.7 %	23	81.3 %	23	100 %	11	43.7 %	18	74.2 %
Skilled manual workers	8	35.3 %	15	64.7 %	10	82.5 %	11	64.6 %	9	63.8 %
Unskilled manual workers	13	34.6 %	10	65.4 %	10	100 %	6	58.6 %	8	99.5 %
Retired	2	1.2 %	11	98.8 %	10	99.4 %	4	47.5 %	6	48.2 %
Unemployed	2	40.8 %	9	59.2 %	9	100 %	4	62.3 %	6	63.6 %
Housewife	16	21.4 %	66	78.6 %	56	83.9 %	26	43.3 %	33	46.5 %
Missing	1		1		0		0		1	
Sector										
Urban	25	26.5 %	65	73.5 %	57	89.0 %	31	47.6 %	40	62.6 %
Rural	20	22.6 %	68	77.4 %	61	90.8 %	32	49.6 %	41	61.3 %
Estate	11	46.4 %	13	53.6 %	12	89.4 %	08	59.4 %	10	75.8 %
Income Category (Monthly Income)										
< Sri Lankan Rupees 10,000	16	23.4 %	37	76.6 %	33	99.5 %	13	35.8 %	18	52.0 %
Sri Lankan Rupees 10,000 to 30,000	32	24.4 %	78	75.6 %	70	89.6 %	44	59.9 %	50	66.9 %
> Sri Lankan Rupees 30,000	5	9.8 %	29	90.2 %	26	85.1 %	14	40.6 %	23	66.7 %
Missing	3		2		1		0		0	
Social status index										
1^st^ quintile (richest)	8	18.2 %	39	81.8 %	33	92.5 %	20	43.2 %	23	60.8 %
2^nd^ quintile	13	28.2 %	38	71.8 %	37	99.8 %	13	30.1 %	18	37.7 %
3^rd^ quintile	14	23.9 %	31	76.1 %	25	79.0 %	20	69.2 %	23	66.5 %
4^th^ quintile	12	21.4 %	30	78.6 %	27	92.0 %	14	57.3 %	22	81.0 %
5^th^ quintile (poorest)	9	53 %	8	47 %	8	100.0 %	4	51.5 %	5	60.9 %
Unsatisfactory Basic Needs Index										
1 (poorest)	6	99.1 %	2	0.9 %	2	100.0 %	1	50.8 %	1	50.8 %
2	5	94.9 %	8	5.1 %	8	100.0 %	6	82.6 %	7	90.6 %
3	8	15.1 %	20	84.9 %	16	81.9 %	12	60.2 %	11	56.0 %
4	17	28.4 %	47	71.6 %	44	99.6 %	19	52.5 %	31	67.3 %
5 (richest)	20	22 %	69	82 %	60	91.0 %	33	41.1 %	41	61.2 %
Place of management										
State	-	-	41	23.4 %	33	79.9 %	12	23.7 %	10	9 %
Private	-	-	100	76.6 %	93	93.8 %	56	57 %	77	76.8 %
Missing	-	-	5		4		3		4	

^a^DM, diabetes mellitus

^b^All percentages were weighted values

There were 42 (25.1 %) individuals describing at least one hypoglycaemic attack. One or more microvascular and macrovascular complication was seen in 186 (92.1 %) and 56 (27.7 %) respectively. Table 3 describes distribution of micro and macrovascular diseases by selected socioeconomic characteristics while Table 4 demonstrates the socioeconomic aspects by place of treatment.

**Table 3:** The distribution of micro and macrovascular diseases by socioeconomic characteristics

Socioeconomic characteristic	Complications of diabetes mellitus (n = 202)^a^			
	Microvascular (n = 186)		Macrovascular (n = 56)	
	Number	%^b^	Number	%^b^
Sex				
Male	92	91.0 %	20	12.5 %
Female	94	93.1 %	36	35.3 %
Age (years)				
35 to 39	11	100 %	1	0.8 %
40 to 44	26	99.4 %	2	7.0 %
45 to 49	34	85.6 %	13	42.4 %
50 to 54	34	80.4 %	14	20.6 %
55 to 59	43	100 %	18	35.0 %
60 to 64	38	94.6 %	8	22.3 %
Ethnicity				
Sinhalese	144	93.1 %	37	23.4 %
Tamil	20	97.6 %	15	93.9 %
Muslim	22	69.0 %	4	32.8 %
Education				
No schooling	5	42.4 %	4	58.5 %
Grade 5 or below	29	92.8 %	17	43.2 %
Grade 6 to Grade 10	73	99.4 %	17	24.7 %
Ordinary Level to Grade 12	42	88.4 %	7	11.8 %
Advanced Level and above	28	86.2 %	7	18.5 %
Missing	9		4	
Occupation				
Professional	2	46.0 %	1	34.0 %
Technical & clerical	11	71.6 %	1	0.6 %
Vendors and sellers	31	86.4 %	6	11.2 %
Skilled manual workers	22	99.9 %	2	11.5 %
Unskilled manual workers	22	99.8 %	11	32.9 %
Retired	13	100 %	1	0.7 %
Unemployed	11	100 %	3	21.6 %
Housewife	72	92.1 %	30	37.3 %
Missing	2		1	
Sector				
Urban	83	91.1 %	20	20.7 %
Rural	82	92.2 %	22	24.6 %
Estate	21	85.0 %	14	53.6 %
Income Category (Monthly Income)				
< Sri Lankan Rupees 10,000	47	92.1 %	20	30.4 %
Sri Lankan Rupees 10,000 to 30,000	101	90.4 %	28	22.6 %
> Sri Lankan Rupees 30,000	33	99.7 %	5	1.1 %
Missing	5		3	
Social status index				
1^st^ quintile (richest)	44	95.1 %	14	36.6 %
2^nd^ quintile	48	93.9 %	12	23.8 %
3^rd^ quintile	44	94.7 %	9	15.1 %
4^th^ quintile	35	83.5 %	9	20.2 %
5^th^ quintile (poorest)	15	87.9 %	12	66.9 %
Unsatisfactory Basic Needs Index				
1 (poorest)	6	99.1 %	4	1.4 %
2	12	99.5 %	7	5.0 %
3	26	92.0 %	8	19.7 %
4	58	89.3 %	12	16.5 %
5 (richest)	84	93.5 %	25	34.3 %
Place of management (n = 146)				
State	36	91.8 %	21	50.2 %
Private	94	90.4 %	17	18.7 %
Missing	5		5	

^a^Presence of one or more complications were considered; ^b^All percentages were weighted values

**Table 4:** The distribution of socioeconomic characteristics by place of treatment

Socioeconomic characteristic	State (n = 41)		Private (100)	
	Number	%^a^	Number	%^a^
Sex				
Male	14	16.6 %	50	83.4 %
Female	27	29.2 %	50	70.8 %
Age (years)				
35 to 39	1	47.9 %	5	52.1 %
40 to 44	2	21.5 %	16	78.5 %
45 to 49	6	20.1 %	19	79.9 %
50 to 54	15	20.9 %	21	79.1 %
55 to 59	10	28.1 %	19	71.9 %
60 to 64	7	19.2 %	20	80.8 %
Ethnicity				
Sinhalese	28	21.8 %	84	78.2 %
Tamil	8	97.6 %	3	2.4 %
Muslim	5	34.7 %	13	65.3 %
Education				
No schooling	1	59.0 %	1	41.0 %
Grade 5 or below	11	19.4 %	10	80.6 %
Grade 6 to Grade 10	21	42.4 %	33	57.6 %
Ordinary Level to Grade 12	4	0.6 %	26	99.4 %
Advanced Level and above	2	11.1 %	25	88.9 %
Missing	2		5	
Occupation				
Professional	-	-	2	100 %
Technical & clerical	-	-	9	100 %
Vendors and sellers	2	5.6 %	21	94.4 %
Skilled manual workers	4	35.0 %	11	65.0 %
Unskilled manual workers	3	0.8 %	5	99.2 %
Retired	5	37.4 %	6	62.6 %
Unemployed	4	37.5 %	5	62.5 %
Housewife	23	31.9 %	40	68.1 %
Missing	0		1	
Sector				
Urban	19	28.8 %	44	71.2 %
Rural	15	23.2 %	51	76.8 %
Estate	7	58.1 %	5	41.9 %
Income Category (Monthly Income)				
< Sri Lankan Rupees 10,000	16	31.6 %	17	68.4 %
Sri Lankan Rupees 10,000 to 30,000	20	20.3 %	57	79.7 %
> Sri Lankan Rupees 30,000	4	16.6 %	15	83.4 %
Missing	1		11	
Social status index				
1^st^ quintile (richest)	9	21.1 %	30	78.9 %
2^nd^ quintile	14	44.3 %	23	55.7 %
3^rd^ quintile	5	13.7 %	26	86.3 %
4^th^ quintile	8	15.6 %	18	84.4 %
5^th^ quintile (poorest)	5	61.2 %	3	38.8 %
Unsatisfactory Basic Needs Index				
1 (poorest)	1	49.2 %	1	50.8 %
2	4	40.3 %	3	59.7 %
3	5	30.2 %	13	69.8 %
4	14	16.8 %	32	83.2 %
5 (richest)	17	23.1 %	51	76.9 %

^a^All percentages were weighted values

Those attending the private healthcare sector had significantly higher rates of, poor control, poor compliance and poor monthly follow-ups (p < 0.05). Meanwhile those who are attending the state healthcare sector had higher proportion with already diagnosed hypertension and ischaemic heart disease (22, 53.7 % and 10, 31.3 % respectively) compared to those visiting the private health sector (37, 44.1 % and 4, 3.6 % respectively).

## Discussion

Our findings show patients with diabetes mellitus in a suburban region in Sri Lanka continue to be poorly managed with high proportions having microvascular and macrovascular complications, irrespective of socioeconomic factors. These figures, though high when compared to high income countries [10, 11, 13, 14], appear to be comparable to similar settings in Asia and Africa [21, 22, 27].

All previous studies on the management and complications of diabetes mellitus in Sri Lanka over the last three decades were on hospital attendees and demonstrated poor management with high complications [28–34]. Our survey, on the contrary captures all hospital attendees, non-hospital attendees and irregular hospital attendees, for a condition predominantly managed in the community, and shows that over the last two decades there have been no improvements in the care of patients (in hospital settings) with diabetes mellitus compared to previous studies. This is true across all socioeconomic categories.

The hospital based studies in Sri Lanka reported lesser proportions with macro and microvascular complications compared to our findings since these have failed to capture non clinic attendees and private sector patients [28–34]. Similar to previous studies commonest macrovascular complication is ischaemic heart disease while the commonest microvascular is peripheral neuropathy [28, 30, 31, 33, 47–49].

Past studies on government hospital based populations in Sri Lanka, have highlighted poor management and control of diabetes mellitus among clinic attendees of these hospitals [48–50]. This study further establishes that those who were managed by the state as well as the private sector are poorly managed.

We demonstrate that the screening of diabetic complications is being performed only in a minority even when the disease is well established, irrespective of the socioeconomic background of the patient. The reasons for such low levels of screening are unclear and needs further exploration. The possible reasons include lack of awareness of doctors, moving between doctors, poor record keeping, poor patient compliance, costs of investigations amongst others.

The poor screening is reinforced by the fact that the already diagnosed with diabetes mellitus have high proportions with complication such as diabetic retinopathy; conditions which arise due to prolonged uncontrolled state of the disease [4].

Perhaps the most surprising new finding from our study is that overall poor management and complications of diabetes mellitus seem to cut across all socioeconomic groups and do not appear to show a detectable social gradient. This is in contrast to high income countries, where prevalence, poor management and complications of diabetes show a social gradient with higher proportion observed among the lower socioeconomic groups [10, 11, 13, 14, 17–19]. Our previous publication, meanwhile, showed an inverse social gradient in the prevalence of diabetes mellitus, which is also in contrary to high income settings [37]. These disparities in diabetes mellitus prevalence, management and its compilations may indicate that Sri Lanka is in a transitional stage. Studies from Sri Lanka including the present study, do not show clear evidence of the increased prevalence of diabetes and cardiovascular disease in lower socio-economic groups compared to higher socio-economic groups [37, 49, 51–53]. This contrasts with experience from the UK, Western Europe and North America where a gradient in mortality across socio-economic groups was observed, i.e. poorer groups affected more by ill-health compared to more affluent groups [54–58]. However, a pattern similar to the current situation in Sri Lanka was observed in England and Wales prior to 1960 when poorer social classes had lower risk of death from coronary heart disease than the higher classes [59]. It is therefore hypothesized that Sri Lanka too is undergoing this transition and the current status is a point in time when the gradient in adverse outcomes appears to be equal across socio-economic groups.

Interestingly higher proportion of microvascular diseases was observed among higher socioeconomic groups while higher proportion of macrovascular diseases was seen among the lower socioeconomic groups. A social gradient is only observed for macrovascular complications within sector and income category. These observations may be due to the co-existing socioeconomic inequality of cardiovascular diseases, which requires further investigation.

The estate sector (the most disadvantaged setting in the country) [60], poorest SSI category and the poorest UBNI category, had the highest proportion of poor control, poor follow-up and not on continuous treatment for diabetes mellitus. Macrovascular complications were seen again most among the estate sector, lowest income group and the poorest SSI category.

Access to private healthcare was high for all socioeconomic strata, except those in the estate sector, despite universal free health care at the point of delivery in the country. In most LICs/LMICs and HICs majority with diabetes mellitus accessed the state sector [22, 26, 27, 61, 62]. Possible reasons for accessing the private healthcare sector are due to likelihood of follow-up by same doctor, reduced waiting times, fewer queues and less congestion, easy accessibility to the private sector specialists due to the absence of a referral system and convenience in terms time of consulting without disruption to work.

However those living in the estate sector seem to utilize mostly the state sector. The main reasons likely to be are the economic and geographical constraints in accessing the private healthcare sector. Perhaps surprisingly the prevalence of microvascular complications in this group is not very different to urban and rural sector. However the high proportion of macrovascular complications can be attributed to co-existing cardiovascular diseases.

Diabetes mellitus patients utilising government healthcare appear to be better managed compared to the private sector even though the microvascular complications were equal in both groups. Although in the state sector there are different levels of care, within each level it is likely to be uniform with less variation in care. Staffing is similar at these levels and is from the same pool of people, whereas in the private sector it is more variable. The quality of state sector services are probably better (though it may be lower than the standard care) due to a number of reasons. One reason could be because the staffing of the state sector hospitals and clinics are administratively accountable to minimum standards or record keeping and documentation. In contrast, the private sector has a wide range or personnel who service their needs. They range from part-time government employed staff or those who work as locums. This tends to fragment their services and follow-up as records are rarely kept. The short-term management as evidenced by HbA_1_C levels may therefore appear to be different between the two sectors. However, in the long-term, the difference may be blurred by patients shifting from one to the other sector and therefore microvascular outcomes may become similar. Also the participants may make return visits to the government sector for follow-up as it is free. Further investigations are required to explore reasons for these observations. In addition strict adherence to the protocols given by the Ministry of Health, Sri Lanka may also help to reduce the high complication rates [44].

Another important finding of this study was that the undetected diabetes mellitus proportion is decreasing. Previous studies in the country have reported undetected proportions higher than 35 % [51, 52]. In South Asia and Africa regions the undetected proportion exceeds more than half the diabetes population [21, 22]. This can be attributed to the opportunistic screening done in both private and state healthcare sectors. High proportion of newly detected in estates, 5^th^ SSI group (lowest social status index category) and 1^st^ UBNI group (poorest UBNI group) indicates that these groups may get less prospects for opportunistic screening. The estate sector had the highest proportion of newly diagnosed suggesting that screening should be targeted to this population.

In most European and Northern American states, the prevalence of diabetes mellitus and its complications show a social gradient with higher rates in those with lower socioeconomic status, lower income levels and poorer educational achievements [5, 7, 13, 17, 19, 48, 63, 64]. In these HICs the socioeconomic differences often also exist in accessing health care, though there are exceptions [8, 16].

Major limitation of the study was the cross sectional nature to investigate a disease with long-term complications. The potential confounding variables that were not measured were psychosocial work characteristics, psychiatric morbidity and life events is another limitation.

Interestingly our findings suggest a crucial difference with high prevalence of complications and poor control of diabetes mellitus across all socioeconomic strata possibly because of the poor quality of screening and deficiencies in adhering to guidelines and protocols. However the reasons contributing to this merit further study including those of service improvements targeting groups such as estates. In addition, it should be explored why patients are discontinuing treatment and not regularly attending the follow-up clinics.

## Conclusion

The vast majority of patients with diabetes mellitus are poorly managed and most had poor control of the disease. Greater proportion of them has microvascular and macrovascular complications and during the medical management most are not screened for these complications. Although majority of poor management and complications observed in the poor geographical and poor social status groups overall socioeconomic gradient seems to be absent with regard to the management and complications of diabetes mellitus.

## Abbreviations

ABI: Ankle-Brachial pressure Index
ECG: Electrocardiography
HIC: High Income Country
IHD: Ischaemic Heart Disease
LIC: Low Income Country
LMIC: Lower Middle Income Country
PAD: Peripheral Arterial Disease
UBNI: Unsatisfactory Basic Needs Index
